# Direct Writing of Functional Layer by Selective Laser Sintering of Nanoparticles for Emerging Applications: A Review

**DOI:** 10.3390/ma15176006

**Published:** 2022-08-31

**Authors:** Eunseung Hwang, Jungmin Hong, Jonghun Yoon, Sukjoon Hong

**Affiliations:** Department of Mechanical Engineering, BK21 FOUR ERICA-ACE Center, Hanyang University, 55 Hanyangdaehak-ro, Sangnok-gu, Ansan 15588, Korea

**Keywords:** selective laser sintering, nanoparticle, functional layer

## Abstract

Selective laser sintering of nanoparticles enables the direct and rapid formation of a functional layer even on heat-sensitive flexible and stretchable substrates, and is rising as a pioneering fabrication technology for future-oriented applications. To date, laser sintering has been successfully applied to various target nanomaterials including a wide range of metal and metal-oxide nanoparticles, and extensive investigation of relevant experimental schemes have not only reduced the minimum feature size but also have further expanded the scalability of the process. In the beginning, the selective laser sintering process was regarded as an alternative method to conventional manufacturing processes, but recent studies have shown that the unique characteristics of the laser-sintered layer may improve device performance or even enable novel functionalities which were not achievable using conventional fabrication techniques. In this regard, we summarize the current developmental status of the selective laser sintering technique for nanoparticles, affording special attention to recent emerging applications that adopt the laser sintering scheme.

## 1. Introduction

Emerging applications such as renewable energy devices [1], flexible/stretchable/wearable electronics [2] and soft robotics [3] are still at their development stages, and discovery of functional smart materials relevant to each application has played a critical role in the advancement of these fields. Introduction of new materials requires concurrent evolution of appropriate processing methods [4,5], discernable from conventional techniques, since the existing technologies are generally designed and optimized for a specific material, i.e., photolithography for silicon wafer, and therefore, are often not compatible with other materials such as chemically synthesized low-dimensional nanomaterials and polymer-based substrates [6]. Among a wide range of processing schemes, the direct writing method, which enables maskless and rapid prototyping, holds great promise, seeing that applications at developmental stages commonly require frequent design changes [7].

Selective laser sintering of functional nanoparticles (NPs) is a representative direct writing method. In a typical selective laser sintering process, a focused laser is utilized as a localized heat source to selectively transform raw material in powder form into a continuous functional layer [8]. An arbitrary patterning is readily accomplished through a scanning procedure, and the feature size can easily reach several microns, allowing high-resolution patterns on-demand. Once the target material is at nanoscale, additional advantages are endorsed from the perspective of material processing. Melting temperature depression observed in ultrasmall sized nanomaterial [9] enables significant suppression of the overall processing temperature. At the same time, the optical properties of the target NP can be fine-tuned [10] with the aim to maximize the absorbance at the wavelength of the laser in use. The combination of these two effects permits energy-efficient sintering of a target NP with minimized heat damage to the underlying substrate, which is important for applications on non-rigid substrates, such as heat-vulnerable plastics and elastomers.

Aided by its strengths, selective laser sintering of NPs has been actively studied in the last two decades, and significant progress has been achieved in terms of the applicable materials and relevant experimental schemes. Based on these advancements, selective laser sintering of NPs has advanced to the rank of mature technologies and is now actively being applied to emerging applications as a supplementary processing method and as a core, indispensable technology. In this review, we briefly summarize the developmental status of selective laser sintering of NPs in terms of applicable materials and experimental schemes, affording special attention to the relevant emerging applications enabled by the selective laser sintering process to discuss the directions of future developments.

## 2. Materials

**Figure 1:**
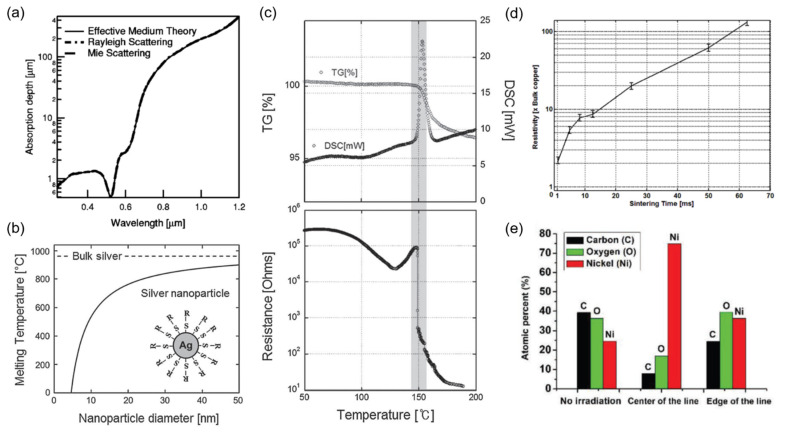
(**a**) Theoretical absorption depth of Au NPs at 5-nm diameter. Reprinted with permission from Ref. [13]; 2003 American Institute of Physics; (**b**) Melting temperature of Ag NP according to diameter calculated from the Gibbs-Thomson equation. (**c**) TGA and DSC measurements of the Ag NP ink at ~5 nm diameter. Reprinted with permission from Ref. [14]; 2011 Wiley-VCH Verlag GmbH & Co. KGaA, Weinheim, Germany; (**d**) Minimum resistivity of laser-sintered Cu electrodes depending on the sintering time. Reprinted with permission from Ref. [15]; 2014 IOP Publishing Ltd., Bristol, UK; (**e**) Chemical composition of NiO NP layer before and after the laser reductive sintering. Reprinted with permission from Ref. [16]; 2019 Wiley-VCH Verlag GmbH & Co. KGaA, Weinheim, Germany.

Due to the unique physical and chemical properties arising from a high surface area and confined size at nanoscale, NPs have been investigated extensively over wide range of scientific areas [11,12]. In this section, we focus on a few types of NPs that draw special attention for selective laser sintering purposes [13,14,15,16] (Figure 1).

### 2.1. Noble Metals

Noble metals, despite their scarcity, have been core materials of interest owing to the excellent stability in ambient conditions and high electrical conductivity. Gold (Au) [17,18,19] has been studied extensively in the early stages in both experimental and theoretical aspects. Absorption depths calculated through different scattering theories [13] suggest that the laser can be utilized as an efficient heating source once the size of the target Au NP is precisely controlled according to laser wavelength. (Figure 1a) Combined with the melting temperature depression phenomenon (Figure 1b), selective laser sintering of Au NP becomes feasible even on heat-sensitive flexible substrates [20]. Analogous ideas have been later applied to different NPs, availing a wide range of materials to laser sintering for flexible and stretchable applications. Early outcomes from laser sintering often show voids at the center of the scanning path [13,21] or an unintended rim [17,22] at the edge due to the thermocapillary force induced by the huge temperature gradient, but such problems can be largely suppressed by controlling solvent evaporation [23]. The excessive thermocapillary force, however, has recently enabled the concept of subtractive laser sintering [24] for highly dense metallic patterns. Molecular dynamic (MD) simulations and experimental studies on lithographically defined Au NPs suggests that the common coalescence time can be in the order of ns after the initial neck growth [25,26,27], yet the characteristic time can be considerably longer given that the number of NP subject to the sintering is much greater in general [28]. The MD simulation also elucidates that other details of the sintering process, which are often difficult to clarify experimentally, e.g., the resultant neck width at different heating rates [26], can be predicted.

As synthesis routes for various silver (Ag) NPs at large quantities have been developed [29], selective laser sintering with Ag NPs has become more common than that of other noble metals [14,30,31]. An Ag NP ink at an average diameter of ~5 nm shows that the melting temperature can be reduced down to ~150 °C as confirmed through thermogravimetric analysis (TGA) and differential scanning calorimeter (DSC) measurements as shown in Figure 1c [14]. Once coupled with low thermal conductivity exhibited by Ag NP compared to its bulk counterpart [32], heat damage can be effectively prevented even on heat-vulnerable substrates [30,33,34] while the resultant electrode exhibits modest robustness against mechanical disturbances [35]. Instead of using presynthesized Ag NP, Ag ion precursor [36,37] or organometallic ink [38] can be employed to achieve laser synthesis and patterning simultaneously. Platinum (Pt), compared to Au and Ag, is not extensively studied [39,40] due to the absence of effective synthesis methods for Pt NP [41]. Recent studies, however, suggest that a Pt layer can be deposited in a precursor liquid environment by laser irradiation to yield outcomes similar to NP sintering [42].

### 2.2. Copper

Among non-noble metals, copper (Cu) [15,43] receives special attention due to its high electrical conductivity compared to that of noble metals, together with superior cost-effectiveness. A critical issue, however, is that Cu is easily oxidized in ambient conditions. As a result, laser scanning speed, which is directly connected to the local heating time, should be carefully optimized [44,45] along with other laser parameters [46], in order to suppress oxidation. More in-depth experiments have revealed that the effect from oxidation becomes significant once the local heating time exceeds ~1 ms as shown in Figure 1d [15]. For a longer heating time, inert gases such as Nitrogen or Argon [47,48] should be introduced during the sintering process to create a highly conductive metallic layer. At optimum conditions, the properties of the resultant Cu layer surpass those created by thermal annealing [49] as confirmed from XRD and XPS analysis [50]. Instead of reducing the local heating time, acid-assisted laser sintering has been developed [51] to remove the oxide layer, and different types of Cu inks [52] are also under investigation for further improvements.

### 2.3. Oxides and Others

There are two different approaches for using metal-oxide NPs in the laser sintering technique, either as the oxide material itself or as a precursor for a conductive layer through a reductive sintering process. ZnO and TiO_2_ are two common oxides that are investigated for sintering processes due to the multiple applications enabled by these materials as functional layers [53,54,55]. Since these oxides possess large band gaps and relatively high melting temperatures, pulsed UV lasers including excimer lasers are widely implemented for efficient sintering, although a CW laser is also a possible option [56]. Upon laser irradiation, the discrete NPs undergo melting and subsequent resolidification steps to change their crystalline structure [53] or phase, which is important for specific applications such as solar cells [54,57]. Direct application of the laser sintering scheme has been also successful with other oxides such as ITO [58], WO3 [59], and more complex oxides [60].

As mentioned, a number of metals suffer from oxidation problems, which become more severe when the material is in NP form that has higher surface-to-volume ratio. Therefore, it is preferable to store an NP in its oxide form and transform it back to its metallic state when needed. A laser-induced photothermochemical reaction enables such reductive sintering of various metal-oxide NPs including CuO [61,62,63,64] and NiO [65,66,67] into their metallic counterparts. (Figure 1e) Through time-resolved normal reflectance measurements, it is suggested that the reductive sintering consists of several steps including densification, reduction and sintering [68]. During the reductive sintering process, slight oxidation can happen at the same time, while a number of intermediate states also can exist [59]. A solvent [61] and capping agent such as polyvinylpyrrolidone (PVP) [16] also play critical role for the corresponding process, acting as both a dispersant and reducing agent.

It should be noted that the range of NPs that is compatible with a laser sintering scheme is continuously growing. Among metals, laser sintering is utilized as a post-process for Al NP slurries to increase the performance of batteries [69]. On the other hand, Zinc (Zn) has been successfully printed and sintered on a bioresorbable polymer substrate through an evaporation–condensation-mediated sintering process [70]. For the improvement of the resultant electrode, nanomaterials with different dimensionality can be mixed with NPs, e.g., Ag nanowire (NW) with Ag NP [71], to create more mechanically robust electrodes, borrowing the idea from steel-wire reinforced concrete. Recent studies include more diverse materials such as liquid metal NP [72], alloy NP [73,74], and coreshell NPs [75] to expand the applications enabled by laser sintering.

## 3. Experimental Schemes

### 3.1. Material Deposition

**Figure 2:**
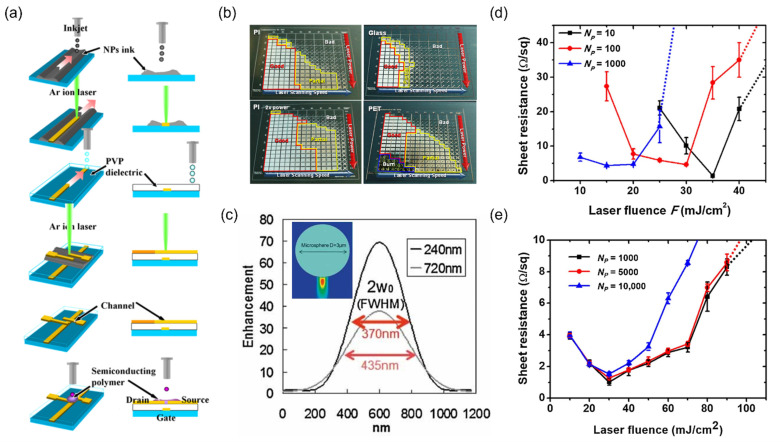
(**a**) Schematic illustration of applying inkjet printing for selective deposition of NP ink, followed by subsequent laser sintering scheme. Reprinted with permission from Ref. [20]. 2007 IOP Publishing Ltd., Bristol, UK; (**b**) Combinatorial study on laser power and scanning speed to find the optimum sintering condition by using galvanomirror scanner. Reprinted with permission from Ref. [28]. (**c**) Submicron focal size created by a microsphere at different gaps. Reprinted with permission from Ref. [81]. 2010 Wiley-VCH Verlag GmbH & Co. KGaA, Weinheim, Germany; Sheet resistance of the laser-sintered Ag NP film by (**d**) nanosecond laser and (**e**) femtosecond laser. Reprinted with permission from Ref. [82]; 2020 Elsevier, Amsterdam, The Netherlands.

In a typical experiment, a target NP is deposited either uniformly or selectively on the substrate. For uniform deposition of the target NP as a nanoscale thin film, spin-coating is commonly used for a lab-scale experiment [30], however, since the unsintered NPs remain on the substrate, the sample generally undergoes an additional cleaning step after laser irradiation using the solvent of the original NP ink. The unsintered NPs can be recycled after the cleaning step, but for minimum use of the NP from the beginning, the NP is deposited only at the designated position using on-demand printing techniques [18,76] as shown in Figure 2a. The initial feature size immediately after the printing process can be as large as ~100 μm [22], but can be reduced down to several microns by using a tightly focused laser beam as the sintering method [28]. Additional templates such as crack-mediated random networks [77] can be implemented to deposit NP ink partially, but where site-selectivity is not important for the target application, target NPs may be provided by different schemes such as aerosol printing [54,78]. To ensure further scalability of the laser sintering scheme, continuous sintering on roll-to-roll-printed Ag NP has been also demonstrated [79,80].

For specific substrates such as polydimethylsiloxane (PDMS), uniform deposition of NP ink is difficult due to their surface properties, and large differences in the mechanical properties between the substrate and the sintered layer also act as obstacles for efficient processing. As a consequence, different experimental configurations including capillary-assisted [83] and shear-assisted [42,84] laser direct writing have been proposed to overcome such limitation. It is further confirmed that a similar scheme is compatible with a wider polymer substrates [85]. On the other hand, NP can be selectively transferred from the donor substrate to the acceptor substrate using a pulsed laser [86,87,88], which is applicable even to arbitrary 3D structures [89]. It has also been shown recently that ultrafast laser heating enables direct 3D assembly and fusion of nanoparticles to create metallic 3D structure at submicron features through ligand transformation [90].

### 3.2. Beam Focusing and Scanning Strategies

A laser beam is often focused and scanned at the same time in laser sintering to create an arbitrary pattern with small feature size. The beam spot size created by a focusing lens is directly related to the size of the laser-induced photothermal reaction [91], and the smallest feature size achievable by a high numerical aperture (NA) lens is in the submicron regime [14]. Scanning can be achieved by moving either the sample by a motorized stage [24] or the galvanomirror combined with f-theta telecentric lens [30] which is compatible with continuous production of conductive film by enabling rapid scanning at meters per second [79] (Figure 2b). On the other hand, throughput can be enhanced by creating a number of beamlets using a microlens array (MLA) [92] or a line beam focus using a cylindrical lens [58]. An MLA can be substituted by a self-assembled microsphere array, which also enables submicron feature size by harnessing near-field characteristics as shown in Figure 2c [81]. For an areal pattern, hatch scanning is inevitable with a spherical or a cylindrical lens, yet a digital micromirror device (DMD) can be implemented as an on-demand digital mask to create a designated pattern instantly, analogous to an ‘optical stamp’ [89,93]. Together with the rapid development of laser sources [94], these studies suggest that the laser sintering scheme has strong potential to be a competent processing technique that enables high-resolution patterning over a large area.

### 3.3. Laser Parameters

Since the physical properties including optical absorbance and thermal characteristics of the target NP are vastly different according to material [95], size [13], and even capping agent [96], selection of adequate laser parameter is a priority [97]. To date, a wide range of lasers at different wavelengths and pulse widths has been successfully implemented as sintering sources, while the details of the resultants may vary. (Figure 2d,e) Since laser sintering is a very complex process that includes a multiphysics problem and various feedbacks between different mechanisms, the optimum laser condition is often found through an experimental parametric study [28,50]. Although it is difficult to understand the exact mechanism behind the sintering process [98], the effects from changing the laser parameters have been investigated in the previous studies. Due to the variation in optical penetration depths, the surface morphologies of the resultant sintered lines as well as the minimum electrical resistivities are different according to the laser wavelength [38,99]. While on the other hand the effect from pulse width is more complex [100]. In terms of processing window, the use of CW laser can be beneficial [101], yet an ultrashort pulsed laser may provide higher conductivity as well as enhanced mechanical properties [82].

## 4. Applications

Until today, it has been confirmed that the laser sintering process can be applied to a myriad of applications that span from common electronic components to unconventional future-oriented devices. In this review, we focus on three different application categories that have recently achieved notable development by adopting the laser sintering scheme.

### 4.1. Electrical Interconnections

**Figure 3:**
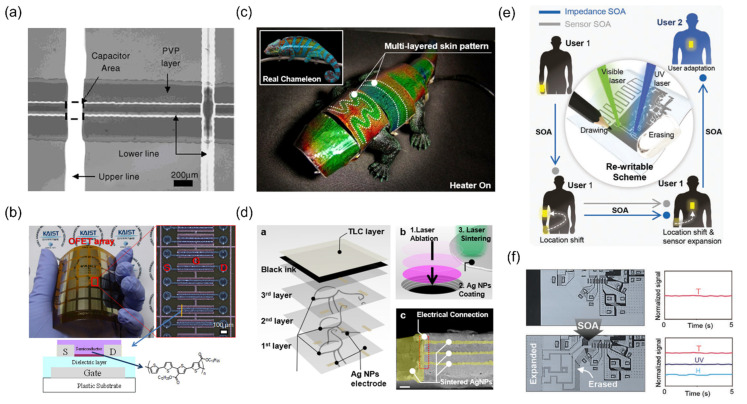
(**a**) Crossover capacitor created by inkjet-assisted laser sintering. Reprinted with permission from Ref. [76]. 2007 Elsevier, Amsterdam, The Netherlands; (**b**) OFET array on PI substrate together with high manification image. (below: cross-sectional structure of a single OFET) Reprinted with permission from Ref. [28]. (**c**) Biomimetic chameleon robot with (**d**) Ag NW and TLC-based Artificial Chameleon Skin. (ATACS) Note that the electrical connections are enabled by the selective laser sintering of Ag NPs, aided by laser ablation of via holes. Reprinted with permission from Ref. [103]. (**e**) Concept of evolvable skin electronics that enables in situ and in operando adaptation (SOA) by re-writable laser processing. (**f**) Addition of UV and humidity sensing capabilities by SOA. Reprinted with permission from Ref [104]. 2022 Wiley-VCH Verlag GmbH & Co. KGaA, Weinheim, Germany.

Selective laser sintering is most intensively studied to create a conductive layer, i.e., electrical interconnections on various substrates, which is crucial for both passive and active electronics. As a fine metallic patterns can be immediately created by the selective laser sintering scheme, photolithographically defined conductive lines can be substituted by the laser-sintered conductive lines. For instance, two parallel metallic microlines created on a highly doped silicon wafer can act as the source and the drain of a transistor [18]. Once a semiconductor material such as air-stable carboxylate-functionalized polythiophene is deposited, it is confirmed that the final device with the laser-sintered lines shows similar performance to the one fabricated with lithographical methods. The corresponding discussion, owing to the highly confined heat-affected zone created by the laser sintering process, can be readily extended to multilayer structures even on flexible substrates. When accompanied by laser ablation process that utilizes large difference in ablation thresholds between sintered and unsintered metal NPs, sharply defined multilayer structure is created without any observable damage on the underlying pattern [102]. Multilayer fabrication capability enables the fabrication of other passive electrical components such as a capacitor (Figure 3a) [76], whereas the reliability of the laser-sintered multilayer has further confirmed in the previous study through the production of 11,520 organic field effect transistor (OFET) on 4-inch wafer size flexible substrate as shown in Figure 3b [28].

While on the other hand, we would like to emphasize that the electrical interconnections created by the selective laser sintering have been applied in two novel applications recently. Firstly, laser-sintered metallic electrode is applied to thermochromic liquid crystal (TLC) based artificial chameleon skin (ATACS) [103] to control multiple heaters separately, which is directly associated to the color and the pattern that the device exhibit. The ATACS is composed of a multilayer structure of colorless polyimide (cPI), Ag NW heater and TLC layer (Figure 3c), and the number of layers increases according to the number of target habitats to blend. In previous studies, laser sintering has been applied only to relatively simple multilayer structures, e.g., transistor that requires two distinct layers with metallic electrodes with an insulating layer in between, but the laser sintering can be applied consecutively to realize a more complex multilayer structure. In this regard, the laser sintering steps are repeated more than three times for the fabrication of ATACS, accompanied by laser ablation process to create via holes, to enable complete electrical interconnections for the final multilayer structure as shown in Figure 3d. The resultant ATACS not only shows clear patterns according to the activation of each heater, but also superior stability towards mechanical disturbances.

Secondly, laser-sintered metallic electrode enables a new concept of evolvable skin electronics, of which system’s impedance and functions can be altered during the operation [104]. The objective of a wearable electronics can be diverse, yet the one of the primary concerns is to measure various physiological data from the body to acquire the current state of the wearer, especially for healthcare purpose. Given that only a single device is used, the conventional wearable device can face the following problems: necessity of new functionality and mismatch of system impedance once the system is altered. The research demonstrates that the combination of laser sintering and ablation of metallic NP using CW laser and pulsed laser (Figure 3e) enables in-situ and in-operando adaptation (SOA) for active, customized wearable devices. By connecting new electronic element via laser sintering, additional measurements, e.g., UV and humidity sensors, become available for the original device (Figure 3f), and it is also confirmed that the system impedance can be optimized according to the body parts, e.g., hand, wrist, chest, where the device is attached. These reports present that the laser sintering scheme is now at a mature technological level and compatible to a complex multilayer structure, while more advanced device concept is realizable, e.g., reprogrammability, once it is combined with other supplementary processes.

### 4.2. Sensors

**Figure 4:**
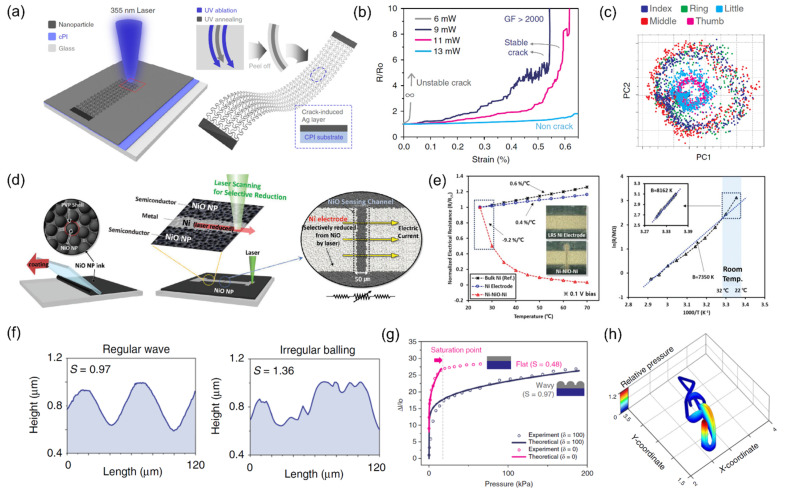
(**a**) Laser-induced crack based skin sensor. (**b**) Strain-dependent resistance of the sensors created at different laser power. (**c**) 2D PCA illustration obtained from the encoding network, showing that finger motions can be identified correctly. Reprinted with permission from Ref. [106]. (**d**) Process illustration for monolithic laser reductive sintering. (m-LRS) (**e**) (Left) Temperature-dependent electrical resistance change of the Ni-NiO-Ni structure and (Right) B-value fitting. Reprinted with permission from Ref [109]; 2019 Wiley-VCH Verlag GmbH & Co. KGaA, Weinheim, Germany; (**f**) Surface profile of the regular wavy structure and irregular balling created at different surface shaping number. (S) (**g**) Resistance responses of flat and wavy structure according to the applied pressure. (**h**) 3D G-clef drawn on the transparent 3D touch device. Reprinted with permission from Ref. [110].

Among diverse sensors, a strain-gauge is often the simplest sensor that consists of metallic strips of known electrical properties under the applied strain. Similar to the aforementioned electrical interconnections, the strain-dependent metallic strip can be directly substituted by the laser-sintered metallic layers [105]. The laser-printed metallic strain gauge responses well to the applied stretch or deflection [70] which is predictable from any other strain sensor. Recent skin sensor demonstrated by Kim et al. [106], on the other hand, proves that the laser sintering possesses great potential to be an efficient manufacturing technique for a next-generation motion sensor that has not been reported before. In this study, Ag NP coated cPI is exposed to UV pulsed laser to complete two different tasks: ablation of cPI and sintering of Ag NP. The resultant is composed of underlying serpentine structure and a crack-induced Ag NP layer. (Figure 4a) The serpentine structure ensures a conformal contact of the sensor with the epidermis accompanied with high stability towards the overall strain, while the cracked layer acts as a highly sensitive strain sensor whose gauge factor can be as large as 2000 (Figure 4b) comparable to other crack-based sensors [107]. Due to its high sensitivity and excellent conformality to the skin, the laser-produced sensor captures previously undetectable signals, which can be decoded to classify various human movements. In particular, the device successfully distinguishes five motions from each finger by attaching a single sensor at the wrist by the aid of a deep neural network, potentiating that the number of sensors required to detect the human motion can be reduced greatly by the simultaneous use of ultrasensitive sensor and machine learning scheme (Figure 4c).

Along with the interests in human-attached sensors to obtain the motion of the wearer, the acquisition of physiological sensor is gaining rapid attention as well due to the rise in the importance of remote healthcare devices for an upcoming aging society. Similar to the discussion above, a change in certain physiological data can be monitored once the physical properties of the sensor are known in advance, but the sensitivity of the sensor is often a problem: a very subtle changes, e.g., temperature variation from exhalation and inhalation of human breathing [108], are often undetectable due to the limited sensitivity. In this regard, Shin et al. proposed an interesting approach to create an ultrasensitive temperature sensor on a flexible substrate monolithically based on the reductive laser sintering scheme [109]. In a typical reductive sintering process, metal oxide nanoparticle is transformed into a continuous metallic layer by scanning the focused laser line by line at a fixed hatch distance. In their study, several scanning lines are skipped intentionally to leave a thin native oxide layer (Figure 4d). The remaining oxide layer, which is NiO in their study, acts as a transition metal oxide channel that shows the characteristics of negative temperature coefficient (NTC) thermistor within the resultant Ni-NiO-Ni heterostructure. Interestingly, the TCR of the resultant temperature sensor is measured to be −9.2%/°C, which yields an extremely high B-value of 8162 K (Figure 4e). It is suggested that such high sensitivity is closely related to various vacancies introduced by the confined photothermal heating.

A touch screen panel is another sensing element that has drawn great attention since the last decade due to rapid increase in the use of portable devices that requires a human–machine interface including mobile phones and tablet PCs. A transparent conductor is a crucial component for the creation of the touch screen panel either for a resistive [30] or a capacitive [111] type, and laser sintering of metal nanoparticle provides an efficient substitute to the conventional ITO-based transparent conductor by forming a regular [30] or a quasi-random [112] metallic grid that is practically invisible to bare human eye. From such simple replacement, huge advancement has been achieved by the recent work by Kim et al. [110]. In their study, the researchers focus on the spontaneous balling effect created upon the laser sintering process, which is often regarded as a metallurgical defect that should be avoided. At a certain laser condition, it is confirmed that regular corrugated structure can be formed (Figure 4f) once the speed of the laser-induced circulating Marangoni flow matches the solidification rate, denoted as a dimensionless number called the surface shaping number. By having well-defined, multiscale metallic structure, the contact area and therefore the electrical pathway changes upon the application of pressure (Figure 4g), which is analyzed to enable the acquisition of pressure information while reading the lateral position as well (Figure 4h).

### 4.3. Heaters

**Figure 5:**
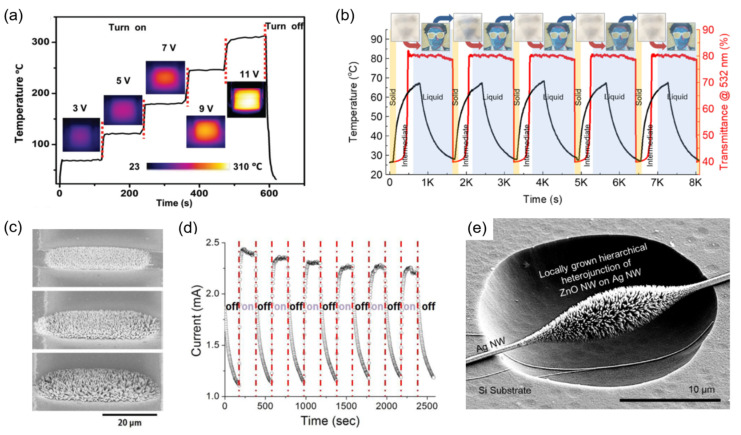
(**a**) Heating characteristics of laser-sintered Ni heater that operates stably at ~300 °C. Reprinted with permission from Ref. [113]. 2021 American Chemical Society, Washington, DC, USA; (**b**) Cyclic operation of the MTC device, showing the durability of the laser-sintered heater. Reprinted with permission from Ref. [114]. (**c**) SEM images of the ZnO NW array hydrothermally synthesized on the laser-sintered Ag NP. (**d**) UV sensor composed of two ZnO NW arrays in contact. Reprinted with permission from Ref. [115]; 2018 Elsevier, Amsterdam, The Netherlands; (**e**) ZnO NW array synthesized on Ag NW connected to the laser-sintered electrical pads. Reprinted with permission from Ref. [116]. 2017 American Chemical Society, Washington, DC, USA.

A heater based on the resistive Joule heating is a component that typically operates under a harsh condition. As a consequence, the robustness of the heater including the stability of the heating electrode at high current and its adhesion to the substrate becomes more important compared to other applications. The laser-sintered metallic layer has been studied extensively especially in the form of a transparent heater [51,112], and special attention has been made to the ones based on a laser-sintered Ni electrode [113] due to its superior thermal stability compared to other non-noble metals. In particular, Nam et al. creates a transparent Ni-based heater on cPI substrate to realize a flexible and transparent heater that aims for high temperature applications. Owing to the outstanding thermal stabilities of both electrode (Ni) and substrate (cPI), the resultant heater operates constantly up to 310 °C while exhibiting rapid heating and cooling characteristics together with excellent mechanical properties. (Figure 5a) As the heaters based on the laser-sintered electrodes become more reliable, they are increasingly applied to proof-of-concept devices such as the recent mechano-thermo-chromic (MTC) device [114] that requires rapid prototyping (Figure 5b).

A more common demonstration coupled to the laser-sintered heater is defrosting or defogging [112], yet the resultant laser-sintered heaters can withstand direct contact to the liquid surrounding. In this regard, the heater based on the laser-sintered electrode has been further applied as the heating source to induce hydrothermal growth at the corresponding electrode to create heterogeneous nanostructure, especially aimed for the synthesis of functional metal-oxide NWs. These attempts can be classified into two categories: in the first, NWs are directly synthesized hydrothermally on the laser-sintered electrode acting as a heating source [115]. It is demonstrated that dense Zinc Oxide (ZnO) NW can be synthesized locally on the laser-sintered electrodes by utilizing them as microscopic heaters (Figure 5c). These NWs expand the functionality of the electrode as shown in the demonstration of a UV sensor composed of two adjacent laser-sintered electrodes which are connected by the hydrothermally synthesized ZnO NW arrays (Figure 5d). On the other hand, laser sintering can be employed to capture another conductive NW, e.g., Ag NW, since the entire sintering process can be easily monitored and controlled at high precision. The captured Ag NW then acts as the template for secondary growth by the aid of electrothermal [117] or photothermal [116] heating (Figure 5e). Through this scheme, the area subject to the NW growth can be reduced down to sub-diffraction regime to enable nanoscale devices.

## 5. Conclusions and Perspective

Selective laser sintering of functional nanoparticles, which has been actively studied in the last two decades, has opened a new route towards facile creation of functional layers. As summarized in this review, major advances have been achieved in every aspect. Starting from noble metals, wide ranges of materials including non-noble metals, metal-oxides and even alloys are now compatible with the laser sintering scheme, enabling the creation of not only simple electrical connections but also active components in ambient condition for more sophisticated, smart devices. The minimum feature size can be reduced even beyond the diffraction limit, whereas continuous efforts are made to increase the overall production throughput by scrutinizing diverse optical schemes, assisted by rapid development in high power laser sources. The laser sintering scheme was first started as a facile substitution for other conventional fabrication techniques, but a number of recent studies reveal that unique morphological and physical characteristics of the resultant often enable rather unexpected breakthroughs to the existing concepts as representatively shown in the examples of the 3D touch screen sensor and the ultrasensitive temperature sensor.

Along with active use of the laser sintering scheme, we predict several future development directions regarding the relevant emerging applications. First, a selective laser process is actively investigated for efficient utilization of other nanomaterials as well, including NWs and 2D materials in particular [118]. A laser is proven to be a useful tool for direct and facile processing of these materials, and a wide range of techniques have been developed, e.g., positioning [119,120], ablation [111], nano-welding [121], pyrolysis [122], modification [123], thinning [124], etc. As a result, the materials which are in the spotlight for next-generation applications are largely compatible with laser processes. Recent studies on the laser process of PI and PDMS, which are common substrates for flexible/stretchable electronics and healthcare devices, further confirm that high-quality micromachining [7,125] as well as adhesive-free bonding [126] between these two substrates are realizable through the laser-induced photothermal reaction. These developmental aspects suggest that laser processes can be among the core fabrication technique for emerging applications, heading towards all-laser fabrication of a device [127,128] up to a system level. We also expect that the laser process will become more valuable as the global semiconductor shortage continues.

Selective laser sintering of nanoparticles can be regarded as a laser additive manufacturing technique, an area that has seen major advancements over the past few years [129,130,131]. As a consequence, the research trends as well as scientific challenges and issues are analogous. Major advantages of additive manufacturing are flexible design and rapid prototyping, and as a result, mechanical metamaterials such as auxetic structures with a negative Poisson ratio [132,133,134,135,136,137,138,139,140,141] can be readily produced and tested by the corresponding technique. Laser-assisted sintering is a highly non-equilibrium process that incorporates a very complicated multiphysics problem with various feedbacks. As a result, in-situ sensing and monitoring of the laser-assisted process [142] are currently subjects of active study, especially to recognize different types of defects in real time. Data-driven optimization of selective laser sintering, e.g., the deep learning approach for tool paths [143], is also becoming popular, and we expect that similar approaches will be investigated for the industrialization of laser sintering processes for nanoparticles.

## Data Availability

Data sharing is not applicable.
